# Autoimmune Pancreatitis Not Otherwise Specified (AIP-NOS): The Importance of Gastroenterology Consultation When Clinical Findings Are Concerning for Pancreatic Cancer

**DOI:** 10.1155/crgm/8916499

**Published:** 2025-09-09

**Authors:** Cooper Alden Josephs, Daniel Mullady, Taylor Templeton-Jager, Nicholas Fuerstenau, Steve Xie

**Affiliations:** ^1^Department of Internal Medicine, Memorial Health University Medical Center, Savannah, Georgia, USA; ^2^Department of Gastroenterology, Memorial Health University Medical Center, Savannah, Georgia, USA; ^3^Department of Clinical Research, Memorial Health University Medical Center, Savannah, Georgia, USA; ^4^Department of Radiology, Memorial Health University Medical Center, Savannah, Georgia, USA; ^5^Department of Pathology, Memorial Health University Medical Center, Savannah, Georgia, USA

**Keywords:** autoimmune pancreatitis not otherwise specified, biliary strictures, obstructive jaundice, pancreatic neoplasms

## Abstract

**Introduction:** Autoimmune pancreatitis (AIP) and pancreatic cancer are top differentials of obstructive jaundice originating from the pancreas.

**Case Description/Methods:** The patient's findings were concerning for malignant biliary obstruction, but a thorough workup determined that the patient had AIP-NOS. She underwent EBS and was discharged on a steroid taper. Follow-up demonstrated complete resolution of symptoms, laboratory markers, and imaging.

**Conclusion:** Adequate pancreatic tissue is not always obtained with 22-gauge needles. Biliary stenting is justifiable in AIP with significant hyperbilirubinemia. It is important to consider AIP for with a pancreatic head mass and obstructive jaundice to optimize outcome.

## 1. Introduction

When patients develop obstructive jaundice originating from the pancreas, autoimmune pancreatitis (AIP) and pancreatic cancer are top differentials. AIP types I, II, and not otherwise specified (NOS) are rare. Its mechanism of hepatobiliary obstruction originates at the molecular level where an immune-mediated inflammatory process targets pancreatic parenchyma and ducts [1]. Unfortunately, differentiating between AIP and pancreatic cancer can be very challenging, and a radiological diagnosis impossible, when both present with mass-forming, diffuse enlargement of the pancreatic gland [2]. AIP commonly mimics pancreatic cancer [3–6]. Pancreatic cancer and its treatments have high morbidity and mortality, and the disease is often lethal [7]. On the other hand, complete resolution of AIP is expected with steroids. Misidentifying AIP for pancreatic cancer leads to unnecessary and invasive procedures.

## 2. Case Report

A 55-year-old Caucasian female presented to a referring hospital with intermittent epigastric and upper quadrant abdominal discomfort for 3 weeks with associated jaundice. Her medical history included hypertension, hypercholesterolemia, type II diabetes, hypothyroidism, tobacco use, minimal alcohol use, and no pertinent family medical history. She had a remote history of a cholecystectomy. She had a 10-pound weight loss over three weeks because pain limited her food intake. Her home medications were glimepiride, albuterol, esomeprazole, ezetimibe, levothyroxine, losartan, and rosuvastatin. She was transferred to our institution for gastroenterology consultation.

**Figure 1:**
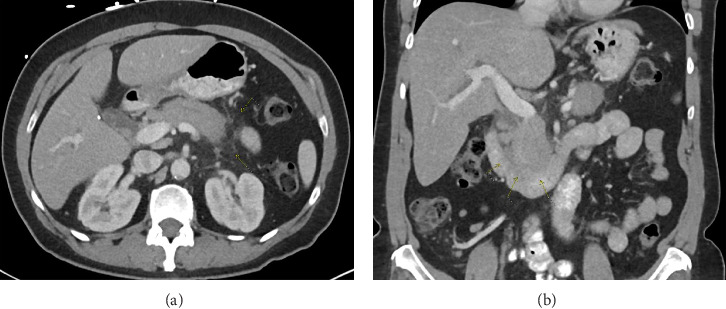
CT abdomen and pelvis. Axial images (a) suggest acute pancreatitis with mild fat stranding surrounding the pancreas, and the coronal images (b) show loss of the interface between the pancreas and the duodenum.

**Figure 2:**
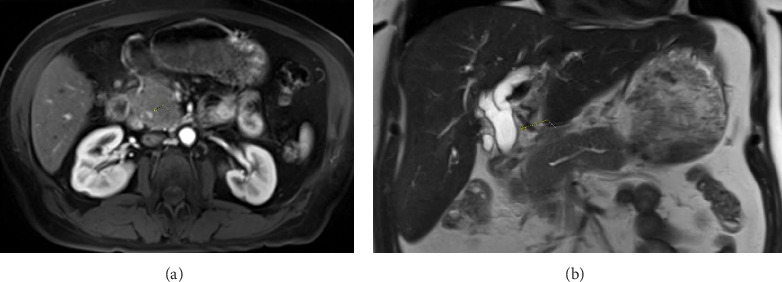
MRI MRCP without contrast. Irregular main pancreatic duct without dilatation (a). Extrahepatic biliary ductal dilatation of the pancreatic head/body fullness (b).

**Figure 3:**
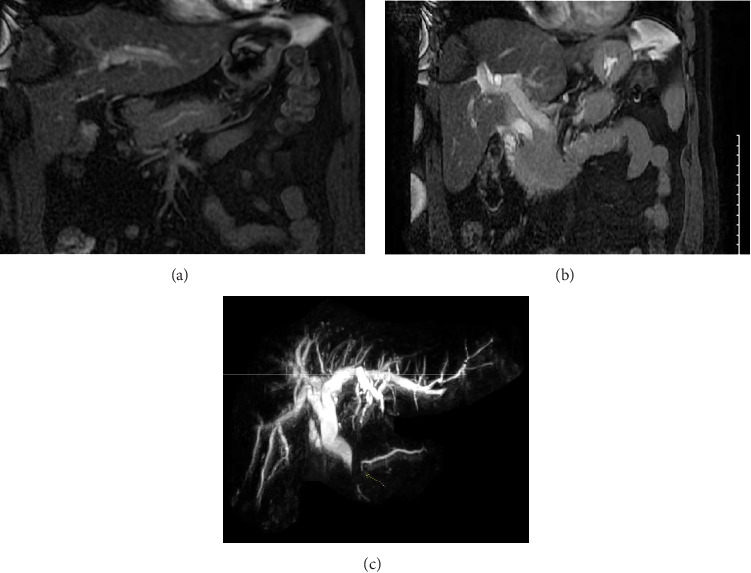
MRI abdomen with and without contrast (a). Focal enhancement along the medial aspect of the distal CBD (b). Abrupt termination of the pancreatic duct signal (c).

Her liver chemistries demonstrated a mixed hepatocellular and cholestatic pattern with an elevated CA 19-9 (194 U/mL). Her lipase, AFP, and serum IgG4 levels were normal. Initial CT scan revealed mild fat stranding surrounding the pancreas, consistent with mild, acute pancreatitis (shown in Figure 1). An MRI MRCP without contrast was performed to further evaluate the hepatobiliary tree (shown in Figure 2). A repeat MRI MRCP with contrast was performed to further evaluate for malignancy (shown in Figure 3). The MRI MRCP was concerning for a mass in the pancreas head/uncinate, and oncology was consulted due to the suspicion for malignancy.

An endoscopic ultrasound with fine needle biopsy (EUS) and endoscopic retrograde cholangiopancreatography (ERCP) with endoscopic biliary stenting (EBS) were performed to evaluate possible pancreatic malignancy and relieve biliary obstruction. During EUS, three passes were made with the 22-gauge biopsy needle using a transgastric approach and using a transduodenal approach. A good visible core of tissue was obtained (NEEDLE BX 22GA ACQUIRE FN ENDO). During ERCP and EBS, one 10 Fr by 7 cm biliary stent with a single external flap and a single internal flap was placed 7 cm into the common bile duct (Boston Scientific STENT BIL 10FR 7CM CNTR BND TEMP RX–S087147298786733). Findings suggested AIP given the characteristic sausage-shaped appearance of the pancreas.

**Figure 4:**
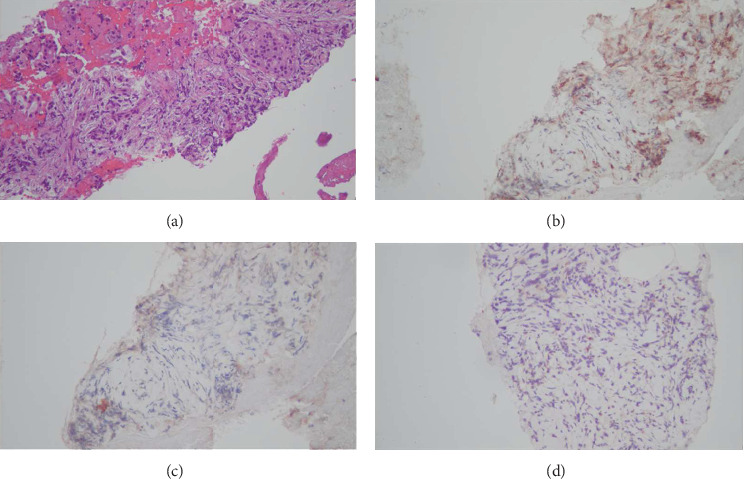
X200 H&E. Pancreatic parenchyma with atrophic pancreatic acinar, increased fibrosis, and infiltrating with small lymphocytes (a). X200 immunohistochemical stain for CD3. CD3 highlights scattered infiltrating T lymphocytes (b). X200 immunohistochemical stain for CD138. CD138 demonstrates absence of plasma cells (c). X200 immunohistochemical stain for IgG4. IgG4 demonstrate absence of IgG4 positive plasma cells (d).

**Figure 5:**
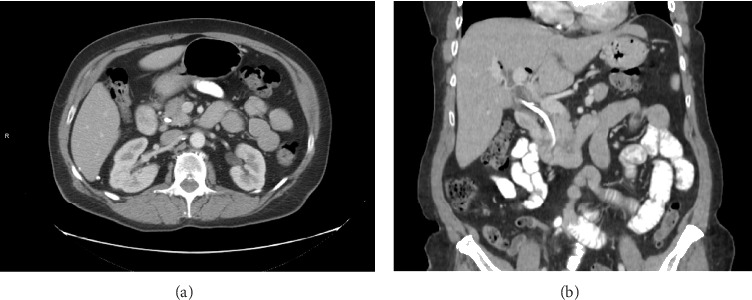
CT abdomen and pelvis. Complete resolution of pancreatic head fullness on the axial image (a) and the coronal image with a CBD stent in place (b).

**Figure 6:**
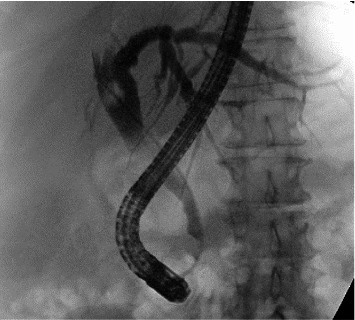
Resolution of bile duct stricture on ERCP fluoroscopy.

Tissue pathology was consistent with chronic pancreatitis without findings of carcinoma. There were scant plasma cells visible, and no samples contained pancreatic ductal tissue. IgG4 staining was negative (shown in Figure 4). Given the EUS appearance and pathology findings, she was diagnosed with AIP not otherwise specified (AIP-NOS). She was discharged on prednisone 40 mg daily for 4 weeks, followed by a 5 mg per week taper, with scheduled outpatient GI follow-up. Repeat imaging 2 months later demonstrated resolution of pancreatic enlargement (shown in Figure 5). Repeat ERCP 3 months after hospitalization demonstrated resolution of the common bile duct stricture, and the biliary stent was removed (shown in Figure 6). She endorsed normal bowel movements at the follow-up visit. She had no symptoms of IBD including no history of bloody diarrhea or chronic diarrhea. She underwent stool-based colorectal cancer screening a year prior and it was negative; she had never had a colonoscopy.

## 3. Discussion

Per AIP guidelines, the patient had AIP-NOS [8]. Our patient fit the AIP-NOS criteria because MRCP findings (appropriate to be used in lieu of endoscopic pancreatogram) demonstrated pancreatic duct segmental narrowing without upstream pancreatic duct dilation, had a sausage-appearing pancreas on EUS, and had significant response to steroids (shown in Figure 3(c)) [9]. Unlike pancreatic cancer of the head, AIP usually shows diffuse pancreatic enlargement, lack of pancreatic duct dilatation, and epigastric pain.

Our case demonstrates the ability of AIP to masquerade as pancreatic cancer given the patient's elevated CA 19-9 and appearance of mass on initial cross-sectional imaging (shown in Figure 3). It is challenging to diagnose AIP because a diagnosis relies on multiple radiologic, pathologic, and laboratory findings [1, 8]. EUS or ERCP alone do not have enough sensitivity or specificity to confirm a diagnosis [10, 11]. CA 19-9 levels were found to be elevated in about half of the patients with AIP who had resection; it is neither completely sensitive nor specific [7].

Unlike previous clinician's success, we did not have success during EUS-guided biopsy with the 22-gauge needles capturing adequate pancreatic ductal tissue (shown in Figure 4) [10]. This would have been helpful to see if the patient had granulocytic epithelial lesions, which is seen in Type II AIP [1, 9]. We decided not to attempt subsequent biopsies with larger needles to try and obtain a definitive tissue diagnosis of AIP. Doing so may have induced pancreatitis or bleeding without necessarily increasing diagnostic yield.

Biliary stenting was justifiable in our case. First, EBS commonly occurs in similar patients to relieve biliary obstruction [5]. Second, our patient had significantly elevated bilirubin greater than 20 times her baseline (10 mg/dL). Most of the participants who only received steroids over EBS in Feng et al.'s study had lower total bilirubin than that of our patient [12]. Third, an AIP diagnosis had yet to be made at the time of EBS.

There can be overlap between the serological, imaging, and histological findings of pancreatic ductal adenocarcinoma (PDAC) and AIP, and it can sometimes be practically impossible to determine whether there is malignancy without surgical resection [7]. There are even some cases where patients had concurrent AIP and pancreatic cancer [13]. Approximately 10% of benign histological pancreatic lesions undergo pancreatectomy, and 30% of these are AIP [7]. Unfortunately, AIP could have been diagnosed before surgery in up to 6.9% of these patients. This author reported that, “the preoperative workup” in a small, yet significant subset of patients was “unsatisfactory,” demonstrating the importance of a gastroenterology specialist consultation [14].

In conclusion, it is important to consider AIP when imaging suggests obstructive jaundice and a pancreatic head mass. The identification and characterization of AIP is relatively new—it was first documented in 1995 [8]. New EUS techniques and diagnostic criteria are in development to increase the sensitivity and specificity of diagnosing AIP [10]. Future research could explore the percentage of patients with pancreatic head masses and obstructive jaundice who undergo preoperative EUS-FNB. Additionally, it could investigate the percentage of patients with a negative initial biopsy who later show benign pathology after resection.

## Data Availability

Imaging was extracted through the hospital's PACS software; laboratory results were extracted from the hospital's EHR (EPIC). Copies are available upon request.
